# Endocytosis‒Mediated Invasion and Pathogenicity of *Streptococcus agalactiae* in Rat Cardiomyocyte (H9C2)

**DOI:** 10.1371/journal.pone.0139733

**Published:** 2015-10-02

**Authors:** Sharma Pooja, Muthuirulan Pushpanathan, Paramasamy Gunasekaran, Jeyaprakash Rajendhran

**Affiliations:** Department of Genetics, School of Biological Sciences, Madurai Kamaraj University, Madurai, 625 021, India; Okayama University, JAPAN

## Abstract

*Streptococcus agalactiae* infection causes high mortality in cardiovascular disease (CVD) patients, especially in case of setting prosthetic valve during cardiac surgery. However, the pathogenesis mechanism of *S*. *agalactiae* associate with CVD has not been well studied. Here, we have demonstrated the pathogenicity of *S*. *agalactiae* in rat cardiomyocytes (H9C2). Interestingly, both live and dead cells of *S*. *agalactiae* were uptaken by H9C2 cells. To further dissect the process of *S*. *agalactiae* internalization, we chemically inhibited discrete parts of cellular uptake system in H9C2 cells using genistein, chlorpromazine, nocodazole and cytochalasin B. Chemical inhibition of microtubule and actin formation by nocodazole and cytochalasin B impaired *S*. *agalactiae* internalization into H9C2 cells. Consistently, reverse‒ transcription PCR (RT‒PCR) and quantitative real time‒PCR (RT-qPCR) analyses also detected higher levels of transcripts for cytoskeleton forming genes, *Acta*1 and *Tubb*5 in *S*. *agalactiae*‒infected H9C2 cells, suggesting the requirement of functional cytoskeleton in pathogenesis. Host survival assay demonstrated that *S*. *agalactiae* internalization induced cytotoxicity in H9C2 cells. *S*. *agalactiae* cells grown with benzyl penicillin reduced its ability to internalize and induce cytotoxicity in H9C2 cells, which could be attributed with the removal of surface lipoteichoic acid (LTA) from *S*. *agalactiae*. Further, the LTA extracted from *S*. *agalactiae* also exhibited dose‒dependent cytotoxicity in H9C2 cells. Taken together, our data suggest that *S*. *agalactiae* cells internalized H9C2 cells through energy‒dependent endocytic processes and the LTA of *S*. *agalactiae* play major role in host cell internalization and cytotoxicity induction.

## Introduction

Group B Streptococci (GBS), or *Streptococcus agalactiae* is considered as a leading cause of life‒threatening invasive bacterial infections in pregnant women, infants, adults and immuno-compromised individuals [1]. These bacteria are Gram‒positive, β‒hemolytic, chain‒forming cocci that are normal residents of the vaginal microflora of 25% of healthy women. GBS causes cellulitis, arthritis and urinary tract infections and is also considered as the most common causative agent of sepsis and meningitis [2‒4]. The *S*. *agalactiae* colonize genital tracts of pregnant women and approximately 50% of newborn babies to these infected mothers are highly susceptible to GBS infection [5, 6].

The molecular events that dictate transition of commensal GBS to invasive pathogens is poorly understood. Successful progression of *S*. *agalactiae* infection requires initial adherence to extracellular matrix followed by entry into the host cells and appropriate expression of virulence gene products in response to the host/external environment during infection [7, 2]. *S*. *agalactiae* produces several virulence factors including pore‒forming toxins, sialic acid‒rich capsule polysaccharide (CPS), C5a peptidase, hyaluronidase and various surface proteins. A total of nine different capsular polysaccharide have been identified and characterized (Ia, Ib, and II-VIII) from *S*. *agalactiae*. Of these, type III serotype is most frequently associated with neonatal infections, whereas other serotypes were known to be involved in infections to adult [5]. *S*. *agalactiae* is also an aggressive infective endocarditis (IE) pathogen, which causes valve damage, heart failure, and thromboembolism [8, 9, 10]. *S*. *agalactiae* colonization in myocardium may lead to infective myocarditis atherosclerosis. Several case reports are available on S. *agalactiae-* induced endocarditis in adults, elderly persons and people with chronic immunosuppressive diseases [9, 11–15]. Only fewer reports were available on acute purulent myocarditis caused by *S*. *agalactiae* [16–18]. *S*. *agalactiae* infection causes high mortality (34–50%) in cardiovascular disease (CVD) patients especially in case of setting prosthetic valve during cardiac surgery [19]. However, there is high risk of *S*. *agalactiae* infection to CDV individuals, the mechanisms underlying the colonization and pathogenesis of *S*. *agalactiae* in cardiac cells has not been well studied.

In this study, we have investigated the pathogenesis of a *S*. *agalactiae* strain isolated from the blood of a valvular heart disease patient [20] in rat cardiomyocytes (H9C2). We found that *S*. *agalactiae* was internalized into H9C2 cells through cytoskeleton‒mediated energy-dependent endocytic processes. Further, Lipoteichoic acid, the cell wall component *S*. *agalactiae*, play major role in internalization and cytotoxicity induction in H9C2 cells.

## Materials and Methods

### Bacterial Strains and Growth Conditions

The *S*. *agalactiae* CVD001A strain was isolated from the blood of a CVD patient and has been previously identified by 16S rDNA sequencing. The 16S rDNA nucleotide sequence of *S*. *agalactiae* CVD001A was available in GenBank under accession number JQ001868.1 [20]. *S*. *gordonii* ATCC 12403 was obtained from American Type Culture Collection (ATCC) and was exclusively used as a non‒invasive negative control to show significant differences in the invasion of *S*. *agalactiae* into the host H9C2 cells. The *Streptococcus* sp. and *Escherichia coli* DH5α strains were grown in Brain Heart Infusion (BHI) and Luria–Bertani (LB) broth, respectively at 37°C overnight with agitation at 200 rpm. Bacterial cells grown to exponential phase were used for infection assays.

### Cell Culture

H9C2 cell line, which was originally derived from the embryonic rat ventricle, was obtained from National Centre for Cell Science, Pune, India. Cells were cultured in Dulbecco’s Modified Eagle’s Medium (DMEM) F12 Ham supplemented with 10% Fetal Bovine Serum (FBS), 100 U ml^-1^ of penicillin, 100 μg ml^-1^ streptomycin and 2.5 μg ml^-1^ of amphotericin B in a humified incubator with 5% CO_2_ and 37°C. Cardiomyoblast differentiation was induced using the method described by Menard *et al*. [21]. Briefly, H9C2 cells were seeded in 75 cm^2^ tissue culture flasks at a density of 3.5 х 10^4^ cells in growth medium. At 50‒60% of confluency, high serum media was replaced by low serum media (1% FBS) and incubated for 4 days in dark with daily supplement of 1 μM All‒*Trans*‒retinoic acid (ATRA) (Sigma, USA). On 5^th^ day of treatment, differentiated cells were trypsinized with 0.025% trypsin‒EDTA solution and seeded for further experiments.

### Bacterial Infection Assay

We evaluated the internalization of fluorescently‒ labeled live and heat‒killed *S*. *agalactiae* into H9C2 cells by live cell imaging using High Content Screening (HCS) system (Operetta, Perkin Elmer, USA). The live *S*. *agalactiae* cells were stained with 5 μg ml^-1^ of acridine orange (AO) in phosphate-buffered saline (PBS) for 5 min at room temperature and washed repeatedly with 1 X PBS to remove the residual stain. Heat‒killed cell suspension of *S*. *agalactiae* was prepared by incubating the cells at 100°C for 15 min and then the viability of heat‒killed cells were assessed by plating on to BHI agar plate. Heat‒killed S. *agalactiae* cells were then stained with propidium iodide (PI) at a final concentration of 48 μM for 5 min at room temperature in dark and washed repeatedly with 1 X PBS to remove residual stain. For infection assay, approximately 1 х 10^4^ differentiated H9C2 cells were seeded in a 96‒well view plate black (Perkin Elmer, USA) in growth medium. The H9C2 cells were infected with fluorescently‒labeled live and heat‒killed *S*. *agalactiae* at different multiplicity of infection (MOI) *viz*., 1:10, 1:100 and 1:1000 in antibiotics and serum free media for 2 h. After infection, the cells were washed five times with 1 X PBS and incubated in their respective media containing 10 μg ml^-1^ of penicillin and 200 μg ml^-1^ of gentamycin to inhibit the growth of extracellular bacteria. The antibiotic concentration used to inhibit growth of extracellular bacterium was based on the determined minimum inhibitory concentration (MIC) for *S*. *agalactiae* and sub‒minimum cytotoxic concentration (Sub-MCC) for H9C2 cells. After incubation, the infected H9C2 cells were washed with 1 X PBS and stained with Hoechst 33342 (5μM) for 30 min at room temperature in dark. Subsequently, the plate was kept in the live cell chamber provided in HCS at 37°C with continuous supply of 5% CO_2_ and the images were captured immediately. In addition, the internalization of live and heat‒killed *S*. *agalactiae* cells into the H9C2 cells were quantified using microtiter plate reader (SpectraMax® M2, Molecular Devices, USA) at excitation wavelength of 502 nm & 535 nm and emission wavelength of 525 nm & 617 nm for AO and PI, respectively. The AO‒stained *S*. *gordonii* ATCC 12403 and non‒pathogenic *E coli* DH5α strain were used as experimental controls for infection assay. All the experiments were performed in triplicates and the mean values were represented with the standard deviation.

### Viable Cell Count Analysis

The intracellular colony forming units (CFUs) from *S*. *agalactiae*‒infected H9C2 cells was enumerated by viable cell count analysis. H9C2 cells were infected with different inoculum of *S*. *agalactiae* (10^4^, 10^5^ and 10^6^ CFU) for 2 h at 37°C in antibiotic free media followed by five times wash with 1 X PBS. The infected H9C2 cells were washed with respective antibiotics in growth medium to remove the extracellular bacteria as described previously. The infected cells were recovered and lysed with 0.1% Triton X-100 (Sigma, USA). The lysate was then transferred to sterile 1.5‒ml microfuge tubes and vortexed vigorously for 10 s to disrupt streptococcal chains and further confirmed by Gram‒staining. Aliquots of the lysates were plated on to BHI agar plates for CFU enumeration. These experiments were performed in triplicates and the mean values were represented with standard deviation.

### Endocytic Inhibition Assay

The uptake of fluorescently‒labeled live *S*. *agalactiae* by H9C2 cells was studied in the presence/ absence of endocytic inhibitor, sodium azide (NaN_3_). H9C2 cells were pre‒treated with NaN_3_ at a final concentration of 10 mM for 1 h prior to the infection. After treatment with NaN_3,_ H9C2 cells were washed repeatedly with 1X PBS for 10 min and suspended in antibiotic free growth medium. The NaN_3_‒treated and untreated H9C2 cells were infected with *S*. *agalactiae* at MOI of 1:100 for 2 h. After infection, the extracellular bacteria were removed and the population of H9C2 cells internalized with *S*. *agalactiae* in the presence and absence of endocytic inhibitor, NaN_3_ were quantified using flow cytometry. Approximately, 50,000 cells were gated on live cells by forward scatter (FSC)/ side scatter (SSC) using fluorescence-activated cell sorter (FACSAria III, Beckton Dickinson, San Jose, CA, USA). The population of H9C2 cells exhibiting green fluorescence for live cell uptake was quantified using FACS at 488 nm employing a blue laser with emission in the 530⁄30 band pass filter. The results obtained were analyzed using FACSDiva version 6.1.3 software package (BD, San Jose, CA, USA).

### Characterization of Endocytic Mechanisms

To further dissect the process of *S*. *agalactiae* internalization into H9C2 cells, we chemically inhibited discrete part of cellular uptake system in H9C2 cells. The different endocytic chemical inhibitors used in this study were: genistein (50‒200 μM), a inhibitor for tyrosine kinases involved in caveolae‒mediated endocytosis [22]; chlorpromazine (20‒50 μM), an inhibitor of clathrin‒mediated endocytosis [23]; nocodazole (5‒30 μM), a microtubule‒disrupting agent [24, 25] and cytochalasin B (50‒100 μM), an actin‒disrupting agent [26]. For endocytic inhibition assay, H9C2 cell were pre‒treated with each endocytic inhibitors for 30 min at 37°C. For microtubule distruption, the H9C2 cells were pre‒treated with nocodazole for 1 h on ice and then warmed to 37°C for 30 min prior to the assay [27]. All infection assays were performed in the presence of each inhibitor at 37°C for 2 h. H9C2 cells treated with each inhibitor without streptococci were used as experimental control. The effect of each inhibitor on the viability of H9C2 cells were studied by trypan blue exclusion assay to ensure that the inhibitor concentration used in this study did not affect the viability of H9C2 cells. The endocytic inhibition of *S*. *agalactiae* in H9C2 cells in presence of different endocytic inhibitors were determined by CFU enumeration. All assays were performed in triplicate and the mean values were represented with standard deviation.

### *S*. *agalactiae*‒Induced Cytotoxicity in H9C2 Cells

*S*. *agalactiae*-induced cytotoxicity in H9C2 cells was determined by confocal microscopy, MTT [3-(4,5-Dimethylthiazol-2-yl)-2,5-Diphenyltetrazolium Bromide] assay and flow cytometry analyses. H9C2 cells were infected with *S*. *agalactiae* for 6 h and morphological changes induced in H9C2 cells by *S*. *agalactiae* infection within 6 h was quantified using Ready-made image analysis tool in Operetta compact high content imaging system (PerkinElmer, Massachusetts, USA). For MTT assay, approximately 50,000 H9C2 cells were seeded per well in 12‒well tissue culture plate and infected with *S*. *agalactiae* at different MOI (1:10; 1:100 and 1:1000) for 2 h at 37°C. After infection, 20 μL of MTT solution (1 mg ml^-1^) was added to each well containing the infected population of cells in 200 μL of growth medium (10% final solution) and incubated at room temperature for 4 h. After incubation, the medium was removed and formazan crystals were dissolved in 200 μL of DMSO. Immediately, absorbance was determined spectrophotometrically at 550 and 690 nm in microtiter plate reader (SpectraMax® M2, Molecular Devices, USA). A subtraction analysis of the dual wavelength (D550 to D690) was performed to increase accuracy of cytotoxicity measurement.

Annexin V-FITC detection kit (Calbiochem, USA) was used to detect *S*. *agalactiae‒*induced apoptosis in H9C2 cells. Approximately 5 х 10^4^ H9C2 cells were seeded per well in 12‒well tissue culture plate and infected with *S*. *agalactiae* at MOI of 1: 100. After infection, H9C2 cells were washed in 1 X PBS and re‒suspended in binding buffer (10 mM HEPES/NaOH, pH 7.4, 140 mM NaCl, 2.5 mM CaCl_2_). Annexin V-FITC was added to the mixture at a final concentration of 100 ng ml^-1^ and incubated in dark at 37°C for 10 min. After incubation, the cells were washed with 1 X PBS and re‒suspended in 300 μl of binding buffer. To this, 10 μl of propidium iodide (PI) was added and subjected to flow cytometry analysis according to the manufacturer’s instruction. The results obtained were analyzed using FACSDiva version 6.1.3 software package (BD, San Jose, CA, USA).

### RNA Extraction and cDNA Synthesis

H9C2 cells were infected with *S*. *agalactiae* at MOI of 1: 100 for 2 h. The total RNA was extracted from the *S*. *agalactiae-*infected and uninfected H9C2 cells using the RNeasy minikit (Qiagen, Germany). The isolated RNA was treated with DNase I (5U/μl) at 37°C for 30 min to remove DNA contamination. The cDNA was synthesized from the purified RNA using Revert Aid First‒strand cDNA synthesis kit (Thermo Scientific, USA) according to the manufacturer’s instructions. The synthesized cDNA was used for further experiment.

### Semi-Quantitative Reverse Transcription (RT)-PCR

The cytoskeleton forming genes, *Acta*1 (Actin, alpha 1, skeletal muscle gene) and *Tubb*5 (tubulin, beta 5 class I) were PCR amplified individually in a 25 μl reaction volume mixture containing 1 X PCR buffer, 2 mM MgCl_2_, 0.8 mM of each dNTPs, 0.1 μM of respective primers and 0.625 U of Taq DNA polymerase using cDNA as template. *Gapdh* (glyceraldehyde‒3‒phosphate dehydrogenase) gene was used as the internal control for all experiments. The list of primers used in RT-PCR experiments are shown in Table 1.The PCR reactions were carried out in thermal cycler (Eppendorf, USA), which consisted one step cycle of 94°C, 30 cycles of 94°C for 30 s, 55°C for 1 min and 72°C for 30 s followed by an final extension at 72°C for 5 min. The amplified products were analyzed on 2% agarose gel.

**Table 1:** List of primers used in the study.

Gene name & Symbol	Primer Sequences (5’-3’)	
	Semi-quantitative RT-PCR	RT-qPCR
Tubulin, beta 5 class I (*Tubb*5)	F: CACAGGTGGCAAGTATGTCC R: GGTCTCATCCGTGTTCTAACC	F: CTGGGACTATGGACTCCGTT R: ACCAACTCAGCTCCCTCTGT
Actin, alpha 1, skeletal muscle (*Acta*1)	F: GACCACAGCTGAACGTGAGA R: GACCACAGCTGAACGTGAGA	F: CCAGAGTCAGAGCAGCAGAC R: CGTGTGGCTCAGTAGGAGAG
Glyceraldehyde-3-phosphate dehydrogenase (*Gapdh*)	F: CGTCTTCACCACCATGGAGA R: CGGCCATCACGCCACAGTTT	F: CACCATCCGGGTTCCTATAA R: AGGGAGGAGCAGAGAGCAC

### Quantitative Real Time‒PCR (RT‒qPCR)

Primer pairs producing ~100‒bp products of *Tubb5*, *Acta 1* and *Gapdh* were designed using Primer Express (version 3.0) software. The lists of primers used in RT‒qPCR are shown in Table 1. The expression levels of *Tubb5*, *Acta 1* and *Gapdh* in *S*. *agalactiae* infected and uninfected H9C2 cells were quantitated by CFX96 real-time PCR machine (BioRad, CA, USA). The assays were performed individually for each genes providing 100 ng of synthesized cDNA as template using Quanti Fast SYBR green PCR kit (Qiagen, Germany). Two independent experiments were performed in triplicate and the gene expression was quantified. The gene expression analysis was performed using relative quantification (ΔΔCT) method. Transcript levels were first normalized to the housekeeping gene, *Gapdh* and expressed as relative level to that of normalized surface. The expression of *Tubb5*, *Acta 1* and *Gapdh* in uninfected H9C2 cells was used as a calibrator to determine the relative gene expression change in *S*. *agalactiae‒* infected H9C2 cells. Nuclease free water was used as no template control (NTC).

### Surface Removal of Teichoic Acid from *S*. *agalactiae* Cells

*S*. *agalactiae* cells were grown in BHI broth at 37°C for 16 h. The grown cells were sub‒ cultured into fresh BHI broth supplemented with different concentrations of benzyl penicillin (0.5, 1, 2, 3 and 4 μg ml^-1^) to remove the surface lipoteichoic acid (LTA). Cells were harvested after 3 h and washed with 1X PBS at 37°C for 5 min. The LTA‒reduced *S*. *agalactiae* cells were used for internalization and cytotoxicity studies. The *S*. *agalactiae* cells grown in the absence of benzyl penicillin was used as experimental control.

### Extraction and Purification of Lipoteichoic Acid

Lipoteichoic acid **(**LTA) was extracted from *S*. *agalactiae* using hot aqueous phenol method [28, 29]. *S*. *agalactiae* cells were grown in BHI broth for overnight at 37°C. The overnight grown bacterial cells were harvested by centrifugation at 10, 000 rpm for 15 min at 25°C. The harvested cells were washed in phosphate buffer (10 mM NaH_2_PO_4_, 1 mM MgCl_2_, pH 7.5) and concentrated to a final concentration of approximately 0.4 g cells ml^-1^. The cell suspension was stirred with an equal volume of pre‒warmed 85% phenol at 65°C for 90 min followed by centrifugation at 10,000 rpm for 20 min at 25°C. The aqueous layer was removed and extracted with an equal volume of chloroform/isoamyl alcohol (24: 1, v/v) followed by centrifugation at 10,000 rpm for 20 min at 25°C and the aqueous layer was retained. The crude extract obtained was treated with RNase (20 μg ml^-1^) and DNase (20 μg ml^-1^) at 30°C for 8 h followed by repeated extraction with cold phenol: chloroform: ethanol (25: 24: 1, by vol.). Finally, LTA was purified on Sephadex gel using fast performance liquid chromatography (FPLC) system (BioRad, CA, USA). The purified LTA was dialyzed against 10,000 volumes of Tris buffer (10 mM Tris/HCl, 1 mM MgCl_2_, pH 7.0 ±5.0, 4°C) for 24 h to remove solvents and nucleic acid fragments. The yield of purified LTA was estimated from chromatographic elution profiles by determining the amount of phosphorus present excluding that ascribed to RNA, and the results were expressed as milligrams of LTA phosphorus per gram (dry weight) of bacterial cells. The dry weight of LTA was calculated from the known phosphorus content of the sample multiplied by a conversion factor (*i*.*e*., 1 mg of phosphorus = 5.81 mg of TA) obtained from the experimentally determined chain length and molar ratio of highly purified TA [30].

### Determination of LTA‒Induced Cytotoxicity in H9C2 Cells

H9C2 cells were treated with different concentrations of purified LTA (*i*.*e*., 50, 25, 12.5, 6.25, 3.13 and 1.56 μg) in growth medium supplemented with 1% FBS and antibiotics for 2 h at 37°C with 5% CO_2_. LTA‒induced cytotoxicity in H9C2 cells were determined by confocal microscopy and MTT assay.

### Statistical Analysis

All experiments were repeated at least three times and the data were expressed as Mean ± SD. Data were analyzed for statistical significance using Student’s unpaired *t*-test and *p*-value of <0.05 was used as threshold for significance.

## Results

### *S*. *agalactiae* Internalization into H9C2 Cells

**Fig 1:**
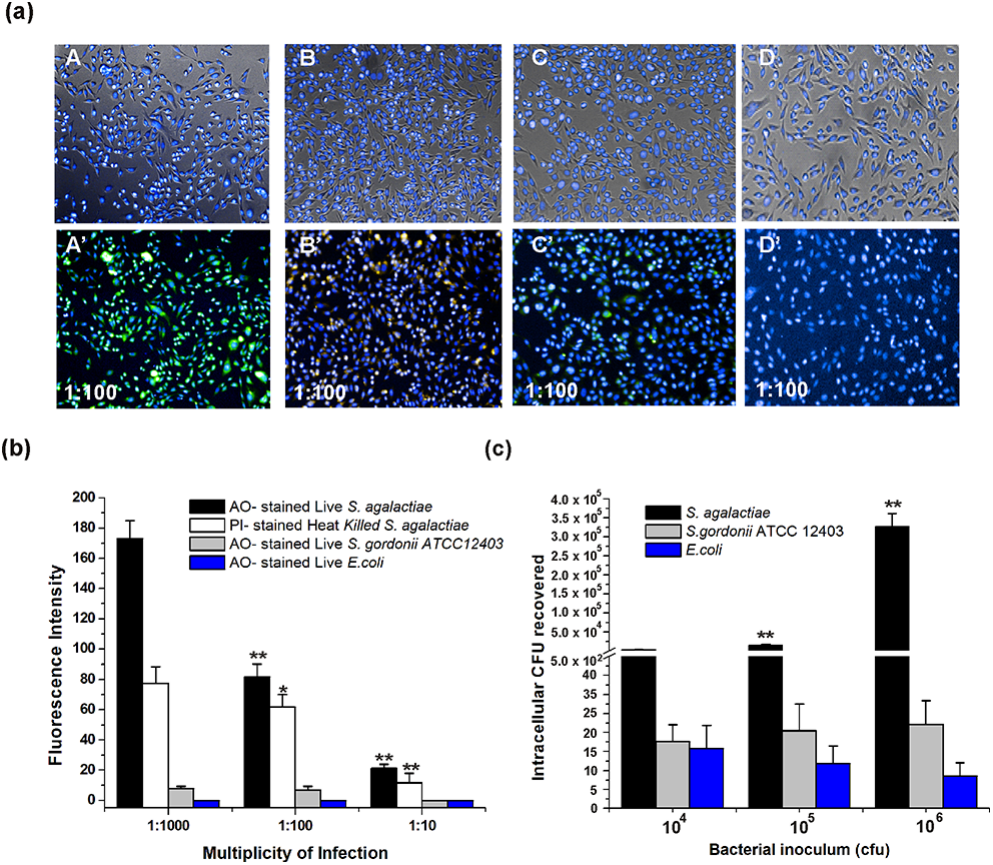
Endocytic uptake of *S*. *agalactiae* by H9C2 cells. (a) Infection assay: A, B, C, D- merged images of bright field and Hoechst 33342 ‒stained H9C2 cells infected with live & heat‒killed *S*. *agalactiae*, *S*. *gordonii* ATCC 12403 and *E*. *coli*, respectively. A’, B’ C’, D’- merged images of H9C2 cells (counter-stained with Hoechst 33342 after infection) and endocytosed fluorescently labeled live‒ & heat‒killed *S*. *agalactiae* (AO & PI‒stained), *S*. *gordonii* ATCC 12403 (AO‒stained) and *E*. *coli* (AO‒stained), respectively. (b) Quantification of endocytic uptake of fluorescently‒labelled live & heat‒killed *S*. *agalactiae*, *S*. *gordonii* ATCC 12403 and *E*. *coli* by H9C2 cells. (c) Viable cell count of bacteria from infected H9C2 cells. H9C2 cells were infected with different inoculum of *S*. *agalactiae*, *S*. *gordonii* ATCC 12403 and *E*. *coli* (*i*.*e*., 10^4^, 10^5^ and 10^6^) for 2 h. The recovered intracellular bacteria from H9C2 cells are represented as CFU/well (Mean ± SD) obtained from three independent experiments. Statistically significant differences are indicated by an asterisk (* *p*<0.05 or **p<0.01).

*In vitro* bacterial infection assay and fluorescence analysis revealed that H9C2 cells could efficiently internalize both live and heat‒killed cells of *S*. *agalactiae* within 2 h of infection. No significant uptake was observed in H9C2 cells infected with *S*. *gordonii* ATCC 12403. However, H9C2 cells could not internalize fluorescently‒labeled live *E*. *coli* DH5α cells even at MOI of 1:1000, incubated for more than 24 h. The endocytic uptake of *S*. *agalactiae* by H9C2 cells was found to be increased with increase in MOI (Fig 1A & 1B). Consistently, the intracellular viable CFUs recovered from *S*. *agalactiae‒*infected H9C2 cells were also found to be increased with increase in MOI. The total intracellular CFUs recovered from *S*. *agalactiae*‒infected H9C2 cells at MOI of 1:10, 1: 100, 1: 1000 were found to be 3.3 x 10^3^, 1.5 x10^4^, and 3.3 x 10^5^ ml^-1^ respectively. Whereas, no significant increase in intracellular viable CFUs were observed in H9C2 cells infected with *S*. *gordonii* ATCC 12403 and *E*. *coli* DH5α (Fig 1C).

### Endocytosis of *S*. *agalactiae* by H9C2 Cells

**Fig 2:**
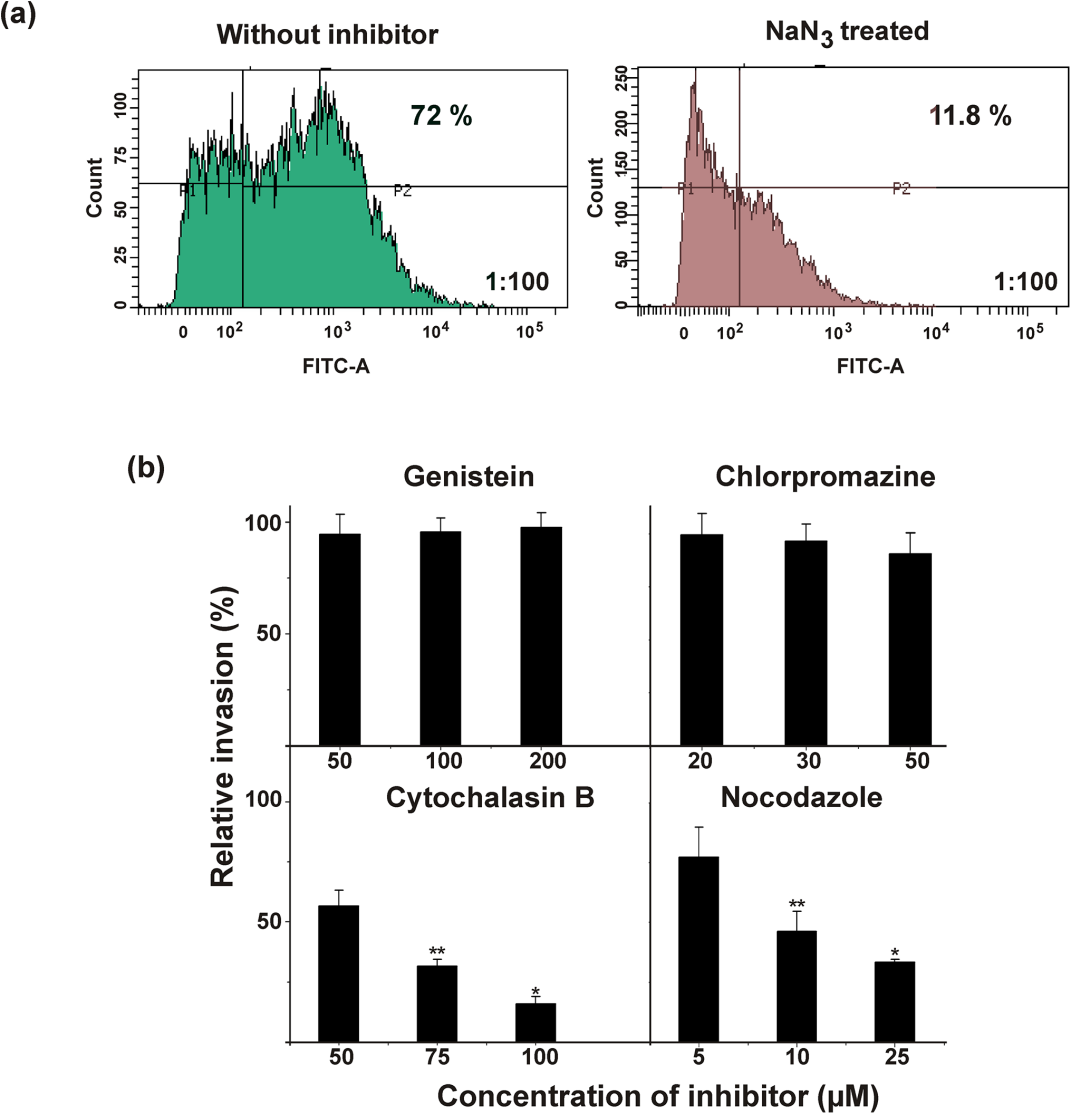
Energy dependent endocytic uptake of *S*. *agalactiae* by H9C2 cells. (a) Quantification of endocytic uptake of fluorescently‒labeled live *S*. *agalactiae* (AO-stained) by H9C2 cells in the presence (green) or absence (brown) of sodium azide by flow cytometry. P1-unstained population of H9C2 cells, P2- population of H9C2 cells internalized with fluorescently‒labelled *S*. *agalactiae*. (b) Determination of intracellular relative viable CFU of *S*. *agalactiae* recovered from H9C2 cells in the presence of different endocytic inhibitors such as genistein, chlorpromazine, cytochalasin B and nocodazole. The results were expressed in relative percentage compared with the experimental control. Statistically significant differences are indicated by an asterisk (* *p*<0.05 or **p<0.01).

Endocytic inhibition studies with sodium azide (NaN_3_) by flow cytometry revealed that after 2 h of infection, 72% of population of H9C2 cells were internalized with *S*. *agalactiae*, whereas only 11.8% the population of NaN_3_ ‒treated H9C2 cells were internalized with *S*. *agalactiae* (Fig 2A). These results suggest that *S*. *agalactiae* could enter the H9C2 cells through an energy‒dependent endocytic mechanism.

Further characterization of endocytic processes of *S*. *agalactiae* in presence of different chemical endocytic inhibitors revealed that the disruption of microtubules and actin formation by nocodazole and cytochalasin B severely impaired *S*. *agalactiae* internalization into H9C2 cells, whereas the *S*. *agalactiae* internalization was not significantly inhibited by chlorpromazine and genistein (S1A Fig). Similar results were observed in CFU enumeration. The intracellular bacterial CFUs recovered from *S*. *agalactiae‒*infected H9C2 cells were drastically reduced with increase in concentration of cytochalasin B and nocodazole. The relative CFU recovered from H9C2 cells for cytochalasin B and nocadazole treatment at their higher concentration were 16% and 33%, respectively. Chlorpromazine and genistein treatment showed no significant decrease in intracellular viable CFUs compared to experimental control (without inhibitor) (Fig 2B). Flow cytometry analysis revealed that 68% population of control H9C2 cells (without inhibitor) was internalized with *S*. *agalactiae*, whereas, only 2.2% and 1.4% population of H9C2 cells were internalized with *S*. *agalactiae* for cytochalasin B and nocodazole treatment, respectively. Consistent with confocal microcopy and CFU enumeration studies, flow cytometry data also showed that *S*. *agalactiae* internalization into H9C2 cells was not inhibited by chlorpromazine and genistein (S1B Fig). All these data suggest that *S*. *agalactiae* internalization into H9C2 cells was mediated by cytoskeleton‒ mediated energy dependent endocytic processes.

### *S*. *agalactiae*–Induced Cytotoxicity in H9C2 Cells

**Fig 3:**
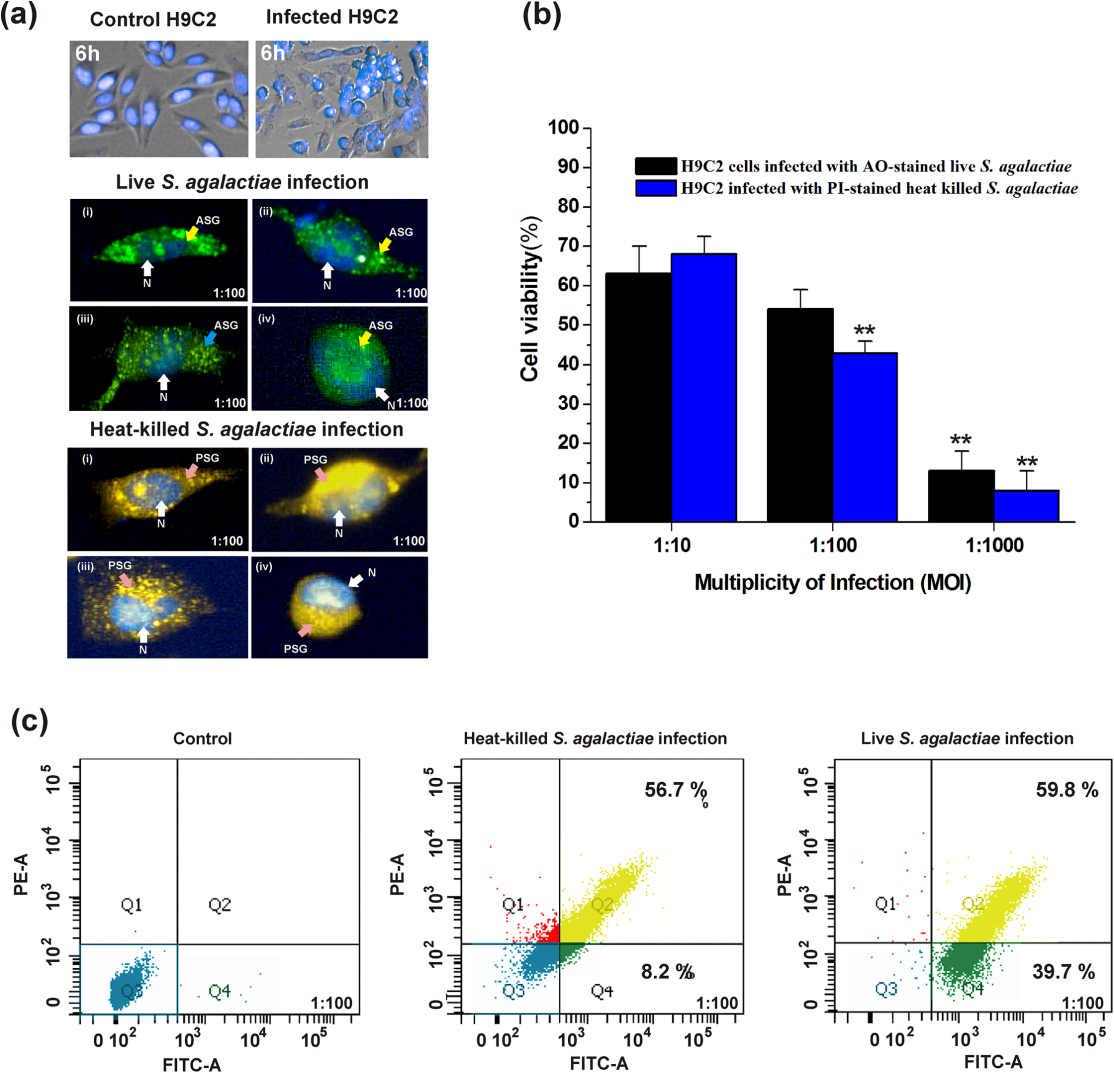
*S*. *agalactiae*‒induced cytotoxicity in H9C2 cells. (a) Morphological changes induced by *S*. *agalactiae* in H9C2 cells after 6 h of infection, Top panel: *S*. *agalactiae*-infected and uninfected H9C2 cells after 6 h of infection. Middle and bottom panel: (i), (ii), (iii) & (iv) are representative confocal micrographs showing changes in H9C2 cell morphology within 6 h of infection with live (ASG) and heat‒killed (PSG) *S*. *agalactiae*. Nuclei (N) of H9C2 cells were shown by Hoechst 33342 staining. (b) Determination of live and heat‒killed *S*. *agalactiae*‒induced cytotoxicity in H9C2 cells by MTT assay. Statistically significant differences are indicated by an asterisk (* *p*<0.05 or **p<0.01). (c) Quantification of live and heat‒killed *S*. *agalactiae*‒induced cytotoxicity in H9C2 cells by flow cytometry. Q3‒live population of H9C2 cells (blue dots); Q4‒early apoptotic H9C2 cells (green); Q2‒late apoptotic H9C2 cells (yellow).

Confocal microscopic analysis revealed that H9C2 cells infected with both live and heat‒killed *S*. *agalactiae* showed changes in morphology within 6 h of infection. The morphological changes were prominent within 6 h in nearly 80% of population of H9C2 cells infected with *S*. *agalactiae*. The representative images for changes in H9C2 cells morphology within 6 h after *S*. *agalactiae* infection are shown in Fig 3A. MTT assay clearly demonstrated that the internalized *S*. *agalactiae* induced dose‒dependent cytotoxicity in H9C2 cells, *i*,*e*., increase in bacterial numbers had led to increased cytotoxicity in H9C2 cells. The maximum cytotoxicity was observed in H9C2 cells infected with live and heat‒killed *S*. *agalactiae* at MOI of 1: 1000 at 2 h (Fig 3B). Flow cytometry analysis revealed that both live and heat‒killed *S*. *agalactiae* induced cytotoxicity in 99.5% (39.5% early apoptotic & 59.8% late apoptotic) and 69.1% (56.7% early apoptotic, 8.2% late apoptotic cells and 4.2% dead cells) of population of H9C2 cells after 2 h post‒infection period (Fig 3C). These results suggested that both live and heat‒killed *S*. *agalactiae* internalization induce cytotoxicity in H9C2 cells.

### *S*. *agalactiae*-Infection Elevates Gene Expression of *Acta*1 and *Tubb*5 in H9C2 Cells

**Fig 4:**
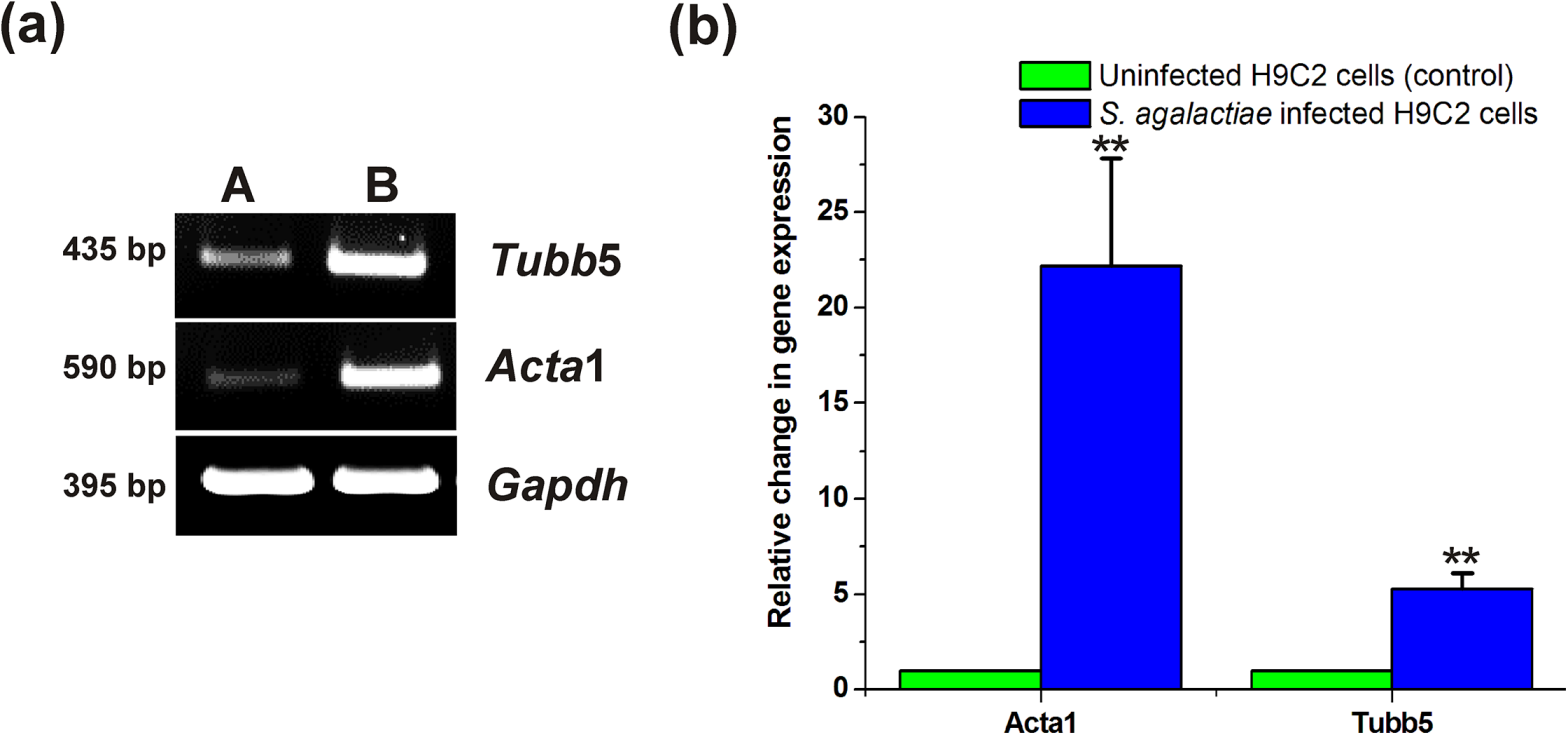
*S*. *agalactiae*‒infection induced gene expression of *Tubb*5 and *Acta*1 in H9C2 cells. (a) Semi quantitative RT-PCR analysis for expression of cytoskeleton forming genes, *Tubb*5 and *Acta*1 in *S*. *agalactiae*‒infected (B) and uninfected (A) H9C2 cells (b) RT Q-PCR analysis for expression of *Tubb*5 and *Acta*1 in *S*. *agalactiae*‒infected and uninfected H9C2 cells. Gene expression values of the uninfected H9C2 cells (control) were set equal to 1 and the relative change in gene expression in *S*. *agalactiae*‒infected H9C2 cells were calculated. RT Q-PCR was performed twice independently and the triplicate measurements of gene expression per gene were included, which yielded similar results. Data shown are the mean relative change in expression ± standard deviation from one such experiment. Statistically significant differences are indicated by an asterisk (* *p*<0.05 or **p<0.01).

Semi quantitative RT-PCR analysis for cytoskeleton forming genes detected relatively higher levels of transcripts for *Tubb*5 and *Acta*1 genes in *S*. *agalactiae*‒infected H9C2 cells compared to uninfected control (Fig 4A). RT Q-PCR analysis revealed that the gene copy number of *Tubb5* and *Acta 1* in *S*. *agalactiae*‒infected H9C2 cells were nearly 5 folds and 22 folds greater than the uninfected control, respectively (Fig 4B). These experimental data provided greater evidence that *Acta*1 is the major factor contributing for *S*. *agalactiae* pathogenesis followed by *Tubb*5.

### Role of Lipoteichoic Acid in *S*. *agalactiae* Internalization and Cytotoxicity

**Fig 5:**
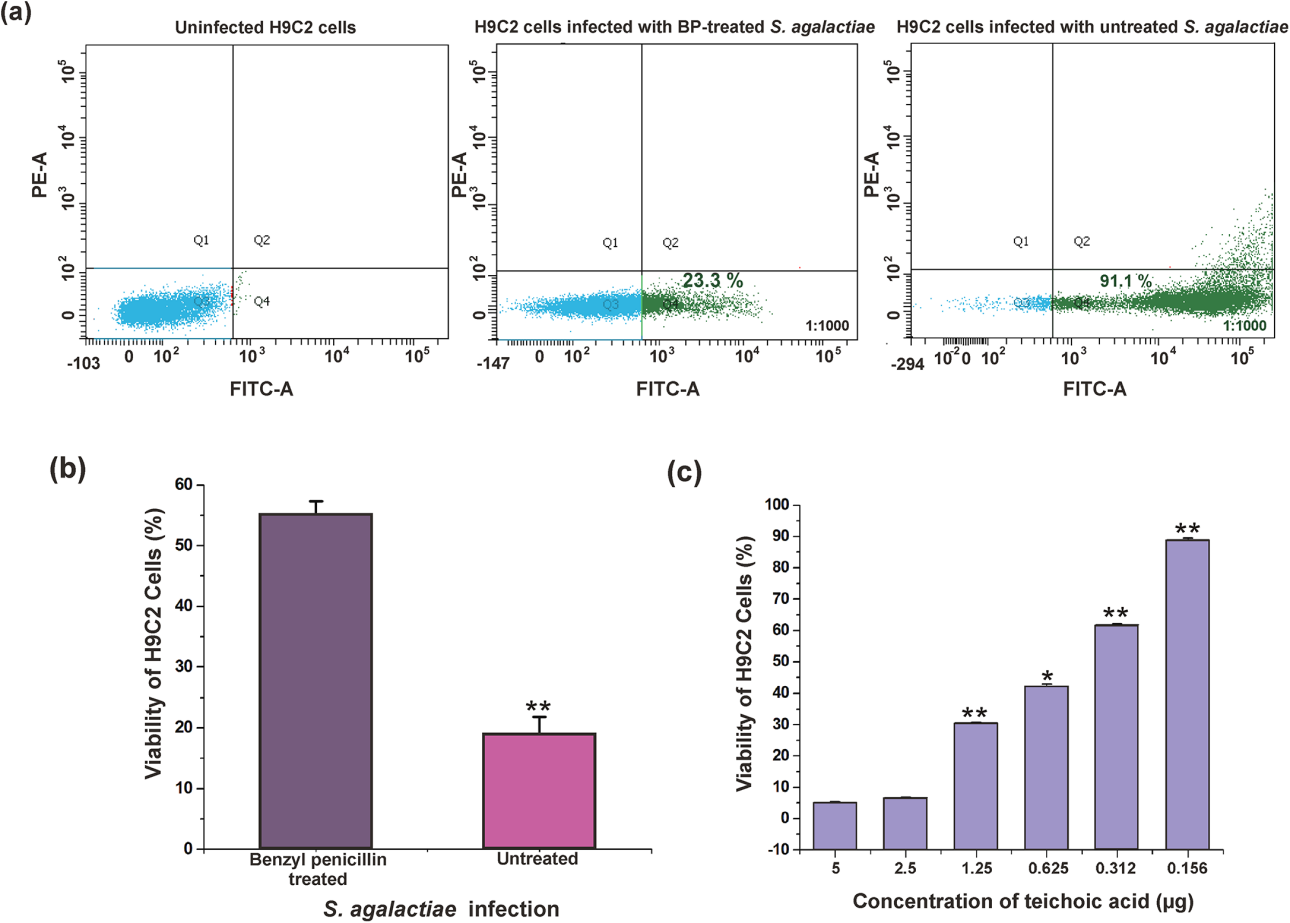
*S*. *agalactiae* LTA‒induced cytotoxicity in H9C2 cells. (a) Quantification of benzyl penicillin (BP)‒treated and untreated *S*. *agalactiae* internalization into H9C2 cells. Q3‒uninfected population of H9C2 cells (blue dots); Q4‒population of H9C2 cells internalized with AO‒stained *S*. *agalactiae* (green dots) (b) Cytotoxicity analysis of BP-treated and untreated *S*. *agalactiae* on H9C2 cells. *S*. *agalactiae* grown in the presence of benzyl penicillin showed reduced cytotoxicity in H9C2 cells compared to untreated control (c) Determination of LTA-induced cytotoxicity in H9C2 cells after 6 h by MTT assay. Statistically significant differences are indicated by an asterisk (* *p*<0.05 or **p<0.01).

Growth studies revealed that *S*. *agalactiae* cells can grow normally up to 2 μg concentration of benzyl penicillin with 52% reduction in LTA (S2A Fig). Infection assay by flow cytometry revealed that untreated *S*. *agalactiae* internalized 91.1% populations of H9C2 cells at MOI of 1:1000, whereas only 23.3% populations of H9C2 cells were internalized with LTA‒reduced *S*. *agalactiae*. Thus, removal of LTA from *S*. *agalactiae* impaired with its ability to internalize into H9C2 cells (Fig 5A). MTT assay also clearly demonstrated that LTA‒reduced *S*. *agalactiae* induced lesser cytotoxicity to H9C2 cells compared to control *S*. *agalactiae* (Fig 5B).

Further, we tested the effect of purified LTA from *S*. *agalactiae* on the viability of H9C2 cells. LTA‒treated H9C2 cells showed morphological changes similar to that of *S*. *agalactiae*-infected H9C2 cells *i*,*e*., detachment of H9C2 cells from the substratum and rounded up within 6 h (S2B Fig). MTT assay also revealed that LTA induced dose‒dependent cytotoxicity in H9C2 cells (Fig 5C). All these data clearly demonstrated that the cell wall component of *S*. *agalactiae*, LTA could play major role in internalization and cytotoxicity induction in H9C2 cells.

## Discussion

Though, *S*. *agalactiae* infections in cardiovascular diseases such as infective endocarditis, myocarditis and pericarditis have been widely reported. However, the association of this bacterium with cardiac cells remains unknown. In the present study, we have used H9C2 cell line as a model system to study the *S*. *agalactiae* pathogenesis, as it is being a surrogate for cardio myoblast and it has the properties of both skeletal and cardiac muscle cells. H9C2 cells can be easily differentiated into cardiac muscle cells by reducing the serum level with the addition of All-Trans Retinoic acid (ATRA) [21, 31]. Differentiated H9C2 cell line also has an advantage of being animal-free alternative and accurately mimics the cellular responses of human cardiomyoctes cell lines. It is also proven to be an excellent *in vitro* model system for prospective molecular studies in heart development and disease [32]

Ability of *S*. *agalactiae* to adhere and invade normal myocardium serves as the primary mechanism in bacterial myocarditis. Here, we have presented the evidence for the internalization and cytotoxicity induction by *S*. *agalactiae* in H9C2 cells. We have shown that both live and heat‒killed *S*. *agalactiae* internalized into H9C2 cells through energy‒dependent endocytic processes. The chemical endocytic inhibitors specific for discrete eukaryotic cell functions were used to ascertain the specific host endocytic mechanism involved in *S*. *agalactiae* internalization. The experiments performed with cytochalasin B and nocodazole have demonstrated the requirement of functional cytoskeletal elements (microfilaments and microtubules) for *S*. *agalactiae* internalization into H9C2 cells. Transcript profiling of genes responsible for formation of cytoskeletal elements also showed elevated gene expression of *Acta* 1 and *Tubb*5 in *S*. *agalactiae*‒infected H9C2 cells, which further substantiated the role of actin and tubulin microfilament in *S*. *agalactiae* internalization into H9C2 cells. Consistent with our findings, earlier study has also shown that viridans group streptococci can enter the human cardiomicrovascular endothelial cells (HCMEC) through the polymerization of actin filaments [33].

The streptococci internalization has also been reported to induce apoptosis in human epithelial cells through caspase dependent pathways [34]. Ulett *et al*. [35] have reported that *S*. *agalactiae* induced apoptosis in murine and human macrophages. It is interesting to note that heat‒killed *S*. *agalactiae* also induced cytotoxicity in H9C2 cells, which suggest that the not only live replicating cells, deads cells can also internalize and induce cytotoxicity in cardiac cells. Earlier report has shown that the administration of heat‒killed *S*. *agalactiae* caused rapid death of adult rats [36]. Heat‒killed *Streptococcus suis* type 2 strain also shown to induce the production of pro-inflammatory cytokines, tumor necrosis factor alpha (TNF-a) and interleukin-6 (IL-6) in murine macrophages [37].

Further, we attempted to investigate the molecular determinant responsible for pathogen internalization and cytotoxicity induction. We found that the cell surface component of *S*. *agalactiae*, LTA play a major role in *S*. *agalactiae* internalization and cytotoxicity induction. Teichoic acid (TA) is a ubiquitous antigenic component and virulence factors of various Gram‒positive bacteria including groups A to G streptococci [38, 39]. These are phosphate-rich molecules found in a wide range of Gram‒positive bacteria. There are two types of TAs: the lipo-TAs (LTAs), which are anchored to the plasma membrane and extend from the cell surface into the peptidoglycan layer; and the wall TAs (WTAs), which are covalently attached to peptidoglycan and extend through and beyond the cell wall. LTA is heat‒stable component of cell membrane and wall of most Gram‒positive bacteria [40, 41]. In our study, the removal of LTA from *S*. *agalactiae* impaired with its ability to internalize into H9C2 cells. Therefore, we believe that heat‒stable LTA is the target molecule involved in internalization of heat‒killed *S*. *agalactiae* into H9C2 cells as well. Previous studies have shown that LTA is well-known ligand for pattern recognition receptors such as, Toll‒like receptors (TLRs) [42]. LTA is a central inducer of inflammatory responses caused by Gram‒positive bacteria, which occurs mainly through the activation of TLR2. LTA can be efficiently internalized into human monocytes and peripheral dendritic cells through receptor‒mediated endocytosis [42, 43]. Earlier reports have also shown that LTA of GBS mediates invasion and induces cytotoxicity in adult & neonatal epithelial cells, HeLa, Giardi heart cells, human embryonic brain and amnion [44, 45]. LTA is involved in signal transduction pathways and induced ERK1/2, JNK, p38, AKT phosphorylation and IL-1 beta gene expression in H9C2 cells [46]. The ability of LTA to modulate host cellular responses might be responsible for induction of cytotoxicity in H9C2 cells. Kristian *et al*., [47] have reported that deletion of *dlt*A gene involved in TA D-alanylation in an invasive GAS diminished its ability to adhere and invade cultured human pharyngeal epithelial cells. Similarly, we also found that LTA‒reduced *S*. *agalactiae* showed lesser ability to internalize and induce cytotoxicity to H9C2 cells. LTA purified from *S*. *agalactiae* also exhibited cytotoxicity in H9C2 cells, which further evidenced the role of LTA in pathogenesis. To conclude, *S*. *agalactiae* could enter the cardiac cells through energy dependent endocytic processes; wherein, the surface determinant of *S*. *agalactiae*, LTA plays major role in *S*. *agalactiae* internalization and cytotoxicity induction in rat cardiomyocytes.

## Supporting Information

S1 Fig Effect of endocytic inhibitors on the endocytic uptake of *S*. *agalactiae* by H9C2 cells. The specific endocytic entry mechanism of *S*. *agalactiae* into H9C2 cells was studied in the presence of different endocytic inhibitors (a) confocal micrographs of H9C2 cells infected with fluorescently‒labeled *S*. *agalactiae* in the presence and absence of different endocytic inhibitors. (b) Quantification of endocytic uptake of live *S*. *agalactiae* (AO‒stained) by H9C2 cells in presence of different endocytic inhibitors such as genistein (violet), chlorpromazine (sky blue), cytochalasin B (brown), nocodazole (yellow) and no inhibitors (dark green) by flow cytometry. P1‒unstained population of H9C2 cells and P2‒ population of H9C2 cells internalized with fluorescently‒labeled *S*. *agalactiae* (AO-stained).
(TIF)

S2 Fig Effect of LTA removal on internalization of *S*. *agalactiae* and the viability of H9C2 cells. (a) Effect of benzyl penicillin on LTA reduction and growth of *S*. *agalactiae* (b) Merged confocal micrographs of bright field and Hoechst 33342‒stained control and LTA‒treated H9C2 cells after 6 h. X and Y represent magnified portion of confocal micrographs of control and LTA treated H9C2 cells, respectively.
(TIF)
