# Supplementary figures and images for: Endocytosis‒Mediated Invasion and Pathogenicity of Streptococcus agalactiae in Rat Cardiomyocyte (H9C2)
Source: PLoS One. 2015 Oct 2;10(10):e0139733. doi: 10.1371/journal.pone.0139733 (PMC4592223; doi:10.1371/journal.pone.0139733)

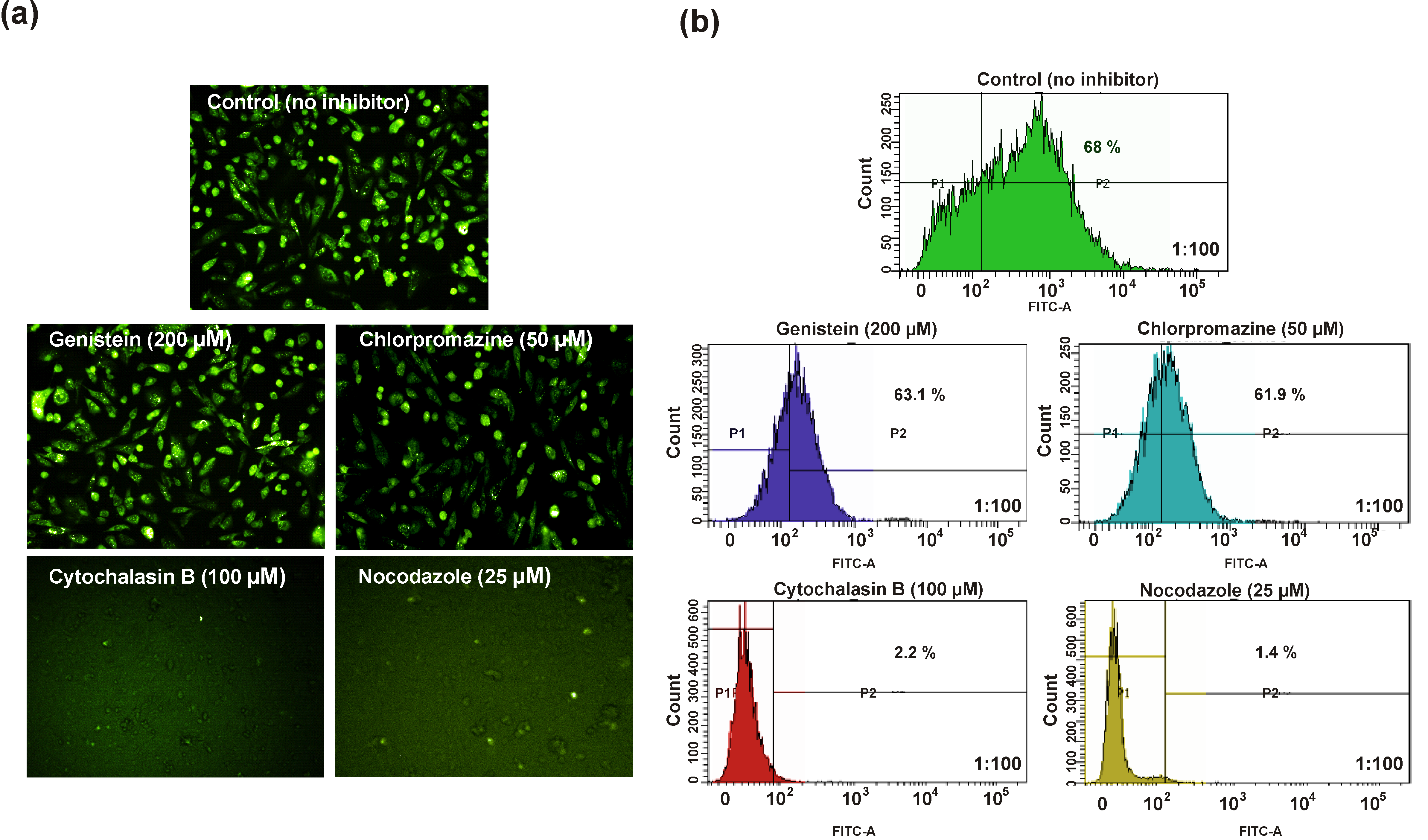

Supplement: S1 Fig — The specific endocytic entry mechanism of S. agalactiae into H9C2 cells was studied in the presence of different endocytic inhibitors (a) confocal micrographs of H9C2 cells infected with fluorescently‒labeled S. agalactiae in the presence and absence of different endocytic inhibitors. (b) Quantification of endocytic uptake of live S. agalactiae (AO‒stained) by H9C2 cells in presence of different endocytic inhibitors such as genistein (violet), chlorpromazine (sky blue), cytochalasin B (brown), nocodazole (yellow) and no inhibitors (dark green) by flow cytometry. P1‒unstained population of H9C2 cells and P2‒ population of H9C2 cells internalized with fluorescently‒labeled S. agalactiae (AO-stained). (TIF) [file pone.0139733.s001.tif]

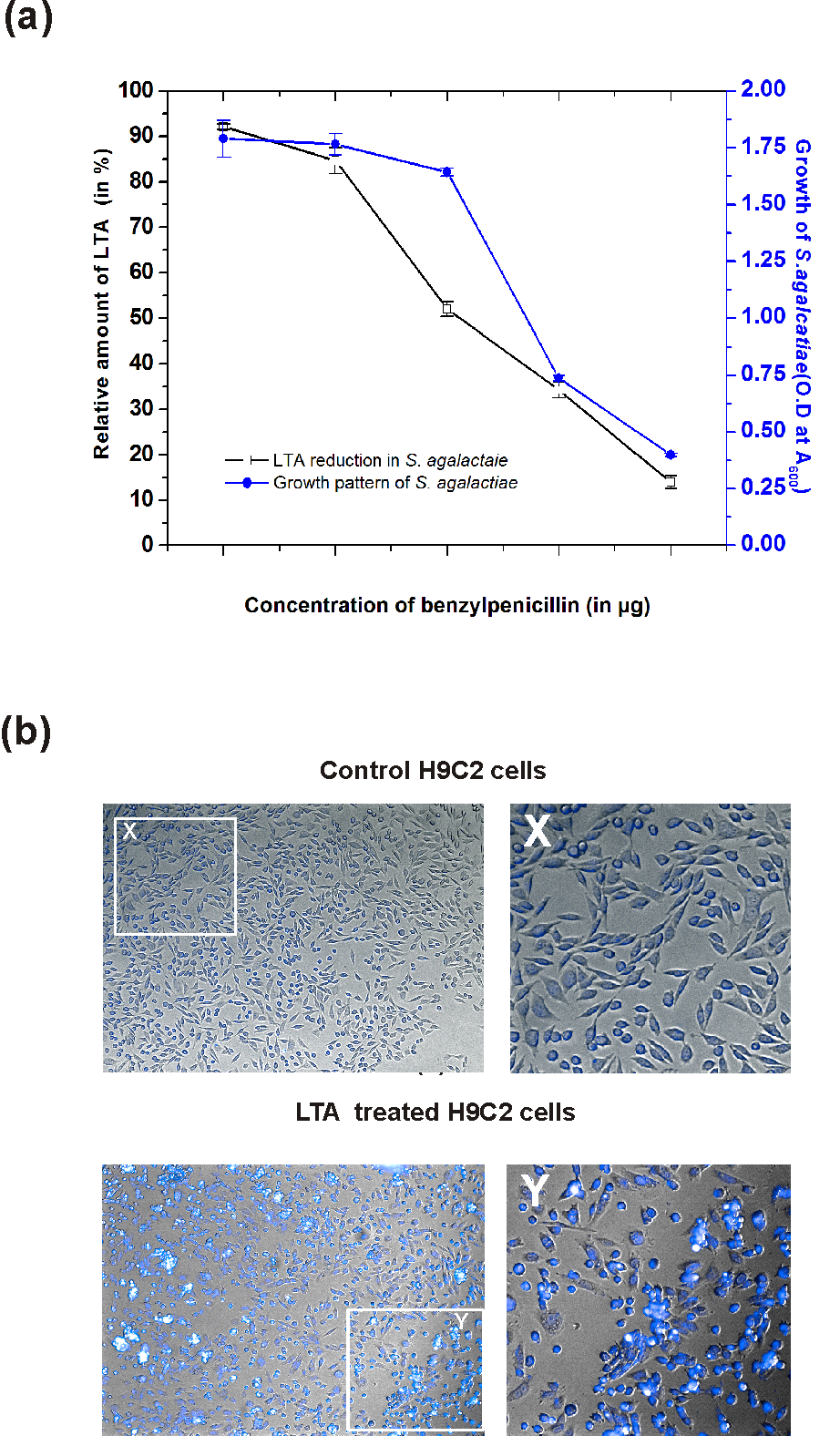

Supplement: S2 Fig — (a) Effect of benzyl penicillin on LTA reduction and growth of S. agalactiae (b) Merged confocal micrographs of bright field and Hoechst 33342‒stained control and LTA‒treated H9C2 cells after 6 h. X and Y represent magnified portion of confocal micrographs of control and LTA treated H9C2 cells, respectively. (TIF) [file pone.0139733.s002.tif]
